# Follow‐Up Adherence After Treatment With Curative Intent for Stage II and III Colorectal Cancer Patients

**DOI:** 10.1002/cam4.70667

**Published:** 2025-02-27

**Authors:** Tara C. Boute, Rik van Eekelen, Marloes A. G. Elferink, Lissenberg Witte Birgit, Johannes H. W. de Wilt, Geraldine R. Vink, Marjolein J. E. Greuter, Veerle M. H. Coupé

**Affiliations:** ^1^ Department of Epidemiology and Data Science Amsterdam Public Health Research Institute, Amsterdam UMC, Location Vrije Universiteit Amsterdam the Netherlands; ^2^ Department of Research and Development Netherlands Comprehensive Cancer Organisation Utrecht the Netherlands; ^3^ Department of Surgery Radboud Institute for Health Sciences, Radboud University Medical Center Nijmegen the Netherlands; ^4^ Department of Medical Oncology University Medical Center Utrecht, Utrecht University Utrecht the Netherlands

**Keywords:** adherence, CEA, colorectal cancer (CRC), follow‐up

## Abstract

**Introduction:**

After colorectal cancer (CRC) treatment, patients undergo five‐year follow‐up involving carcinoembryonic antigen (CEA) tests, imaging, and colonoscopies. This retrospective cohort study explores adherence to the CRC follow‐up guideline in the Netherlands until 2021 and its association with treatment of recurrences with curative intent.

**Methods:**

Stage II/III CRC patients with recurrent disease within 3 years after diagnosis were selected from the Netherlands Cancer Registry (*n* = 430). Adherence to CEA tests, imaging, and colonoscopy was classified as ‘according to/more follow‐up’ or ‘less follow‐up’ than recommended. Logistic regression analyses examined factors associated with receiving less follow‐up and the relationship between ‘follow‐up adherence’ and ‘treatment with curative intent’, potentially mediated by ‘mode of detection’ (symptomatically vs. routine test).

**Results:**

In total, 18.3% patients had fewer CEA tests, 41.4% fewer imaging, and 56.1% fewer colonoscopies than recommended. Factors associated with fewer follow‐up moments were tumor localization, age (≥ 75 years), comorbidities, tumor differentiation and adjuvant chemotherapy. Patients receiving fewer CEA tests faced 4.8 times higher odds (95% CI: 2.9–8.1) of symptom‐detected recurrence and were less likely to be curatively treated (OR = 0.5; 95% CI: 0.3–0.9). Mediation analysis indicated a significant average causal mediation effect (*p* = 0.003), emphasizing the mediating role of mode of detection. Receiving fewer imaging and colonoscopies showed insignificant total effects on treatment with curative intent.

**Conclusion:**

Our findings offer insights into follow‐up adherence, detection mode, and treatment with curative intent. The discovery that adherence was highest for CEA, along with the correlation between CEA adherence and treatment with curative intent, aligns with the recent adaptation of guidelines emphasizing CEA measurement over imaging.

**Abbreviations**
ACME: average causal mediation effects
ADE: average direct effects
CEA: carcinoembryonic antigen
CI: confidence interval
CRC: colorectal cancer
HR: Hazard Ratio
MAR: missing at random
NCR: Netherlands Cancer Registry
OR: Odds Ratio
OS: Overall Survival
TNM: tumor‐node‐metastasis

## 1 Introduction

The cure rate of colorectal cancer (CRC) has increased in the last decade due to the implementation of screening programs and better treatment options, but approximately 20%–30% of patients with stage II/III CRC develop a recurrence [1, 2]. A general assumption is that early detection of a recurrence increases the chance that the recurrence can be treated curatively, which is the rationale for follow‐up of patients after resection. Although follow‐up is recommended in the guideline, benefits of intensive follow‐up have not been unequivocally established.

In the Netherlands, stage II/III CRC patients are recommended 5 years of follow‐up after surgical resection, as most recurrences occur within that time period [1, 2]. This follow‐up is usually organized within the hospital [3]. Until the end of 2020, the protocol included a colonoscopy 1 year after surgery, an ultrasound or CT‐scan from the abdomen twice per year for the first two years after surgery and carcinoembryonic antigen (CEA) measurements every 3–6 months for the first three years and every half year thereafter. In 2021, the guideline for the ultrasound or CT were changed to only one CT‐scan performed 1 year after surgery.

The revision of the guideline in 2021 was based on an ongoing debate in the literature about the ideal frequency and intensiveness of follow‐up of stage II/III colon cancer [4, 5, 6]. A Cochrane systematic review from 2016 concluded that intensive follow‐up strategies did not show a significant benefit in terms of Overall Survival (OS) (Hazard Ratio (HR) = 0.91, 95% Confidence Interval (CI): 0.80–1.04) for patients with non‐metastatic colorectal cancer compared to less intensive or standard follow‐up approaches [7]. A recent meta‐analysis also showed no significant impact of intensive follow‐up on OS for CRC (HR = 0.99, 95% CI: 0.92–1.06) based on 8 studies with a low risk of bias [8]. However, when considering all 33 included CRC studies, it suggested improved OS (HR = 0.82, 95% CI: 0.73–0.91) and higher rates of curative intent treatment for colorectal cancer (CRC) recurrences (RR = 1.60, 95% CI: 1.21–2.11) [8]. Additionally, a review by the National Institute for Health and Care Excellence (NICE) has reported a significant difference in OS between “more intensive versus less intensive follow‐up” interventions (HR = 0.89, 95% CI: 0.80–0.99) [9].

The effects of abovementioned reviews suggest at best a small survival benefit of intensive follow‐up regardless of statistical significance. It should be noted that it is challenging to evaluate the impact of follow‐up intensity on survival due to the small expected effect size and high sample size requirements. In addition, the populations in the trials included in the abovementioned reviews do not reflect the general population of patients outside of the context of trial‐based research. The general population tends to be older and have more comorbidities than the population participating in trials, so intensive follow‐up strategies may result in lower adherence. Therefore, it is essential to study the adherence to follow‐up in population based observational data such as cancer registries.

In this study, we determined adherence to the follow‐up guideline in place before 2021 using the observed patterns of follow‐up testing. We subsequently investigated which factors are associated with receiving less follow‐up than the guideline recommends. Lastly, we assessed the association between adherence to follow‐up and the possibility to treat a recurrence with curative intent.

## 2 Methods

### 2.1 Design and Study Population

In this retrospective cohort study, we used data from the Netherlands Cancer Registry (NCR) on stage II/III CRC patients who were diagnosed in Q1 and Q2 from 2015 with a primary tumor in the colon (C18.0, C18.2–7) or rectum (C19.9, C20.9). The NCR contains data from all cancer patients in the Netherlands and data are registered by well‐trained data managers. The Q1 and Q2 2015 cohort consisted of 3204 stage II or III CRC patients of which 690 patients developed recurrent disease within 3 years. Not all necessary patient‐level data on follow‐up visits is routinely registered in the NCR. Additional data was collected from electronic health records following a highly structured protocol to ensure consistency. We sampled 500 patients with recurrent disease from hospitals across the country, as much as funding allowed. We only included patients who developed recurrent disease within 3 years after surgical resection of their primary tumor. We excluded patients with a recurrence within 6 months of follow‐up as we considered this time period too short to accurately determine whether they adhered to recommended follow‐up. For the selected patients, data on follow‐up (visits, test) was collected in 2021 from electronic hospital patient files.

### 2.2 Available Data

For each patient, the type and date of each test during follow‐up was recorded. Also, mode of detection of recurrence was recorded, indicating whether a recurrence was identified based on a test that was scheduled due to symptoms (referred to as interval recurrence) or through diagnostic testing following abnormalities observed at a routine test during follow‐up. Data managers from the NCR retrospectively reviewed patient files to check whether the recurrence was detected through a scheduled follow‐up test or whether an additional consult was requested due to symptoms.

The primary outcome of this study is the intent of treatment of the recurrence, since finding the recurrence early enough to be able to curatively treat it is an important goal of follow‐up. Treatment is considered to have a curative intent if resection and/or local treatment of all recurrent disease has taken place. Patients who had no resection of the recurrent tumor(s) were classified as receiving non‐curative treatment.

The 7th edition of the UICC TNM (tumor‐node‐metastasis) classification was used as reference for pathological tumor stage [10]. Pathological stage was used, except for patients who underwent neoadjuvant treatment or lacked pathological staging data, for whom clinical staging was used instead.

### 2.3 Operationalization of Follow‐Up Adherence

The start of the follow‐up period was defined as the day on which resection of the primary tumor was performed. The end of the follow‐up period coincided with the date of recurrence, as all patients included in the study experienced recurrence. The date of recurrence was determined by confirmation through testing in the hospital.

Follow‐up adherence for CEA, ultrasound/CT imaging, and colonoscopy was assessed per patient. Due to difficulties in distinguishing between scheduled routine versus additional diagnostic tests, we combined the ‘according to guideline’ category with the ‘more than guideline’ category for analysis, simplifying adherence classification to ‘according to or more than guideline’ and ‘less than guideline’. Categories were made separately for each test and for all three tests together, based on the guideline and expert consultation.

For the CEA measurements, the guideline recommended an average of 2 to 4 tests per year for the first 3 years. Because the period of time‐to‐recurrence differed between patients, the total number of tests during follow‐up did not provide an accurate estimation of whether tests were done according to guideline recommendations. We therefore calculated rates per test, dividing the total number of tests by the patients' time in years in follow‐up, to obtain the average number of tests per year. If a patient had less than 2 CEA tests annually, the follow‐up was categorized as ‘less CEA than guideline’.

For the imaging techniques, the guideline recommended CT‐scans and ultrasounds of the abdomen, or CT thorax‐abdomen for rectal cancer patients. We calculated the rates, again by summing all the abovementioned tests and dividing them by time in follow‐up. For the initial 2 years of follow‐up, abdominal imaging should have been conducted twice annually, and subsequently, once per year. Therefore, having fewer than two tests per year was considered ‘less imaging than guideline’. Since all patients developed a recurrence within 3 years, imaging during the third year was not considered.

Lastly, the guideline stated that a colonoscopy should be performed 1 year after surgery. For patients who were not eligible to receive a total colonoscopy, the guideline recommended a CT‐colonography as an alternative. A three‐month buffer range was used; thus, it was assessed whether patients had a colonoscopy or CT‐colonography within the recommended timeframe of 9 to 15 months. Patients who did not undergo one of those tests within this period were labeled as ‘less colonoscopies than guideline’. For patients who developed a recurrence before 15 months, some may not have received a colonoscopy because their follow‐up ended before the procedure could be performed. Consequently, all patients diagnosed with a recurrence within 15 months were excluded from the analyses related to colonoscopy and overall adherence, as accurate classification for these patients was not possible.

### 2.4 Statistical Analysis

Firstly, descriptive statistics were used to give an overview of the population characteristics, including percentages and median estimates with the first and third quartile (Q_1_–Q_3_). Follow‐up adherence was graphically described in histograms. This was done for CEA, colonoscopy, and imaging tests separately, as well as for an ‘overall’ category for all three tests in which patients were categorized as receiving ‘less follow‐up than guideline’ if they were categorized as ‘less than guideline’ for at least one test.

Secondly, the association between patient characteristics and receiving less follow‐up than the guideline was analyzed by means of logistic regression models. The following variables were added to the models for each test, based on literature and input of clinical experts in the team: age, number (No.) of comorbidities, tumor location, stage, tumor differentiation and having received post‐surgical systemic chemotherapy [11].

Thirdly, logistic regression models were used to study the relationship between ‘follow‐up adherence’ and ‘treatment with curative intent’. Less follow‐up than the guideline may delay recurrence diagnosis, affecting curative treatment eligibility. Mode of detection is therefore tested as a mediator between follow‐up adherence and treatment with curative intent for recurrence. More explanation about the method and interpretation of the mediation analyses can be found in file S1.

The “medflex”‐package in R was used to calculate the average causal mediation effects (ACME) and the average direct effects (ADE). We calculated the indirect effect using non‐parametric bootstrapping procedures, because it does not assume specific distributions for the data, and it helps quantifying potential uncertainty around the mediation effect estimates. Unstandardized indirect effects were computed for each of the bootstrapped samples, and the 95% confidence interval was computed by determining the indirect effects at the 2.5th and 97.5th percentiles. The analyses were corrected for age and comorbidities as confounders based on the literature and clinical expertise [11].

Lastly, we performed a sensitivity analysis to assess the impact of a less strict classification of follow‐up adherence to CEA‐tests and imaging on the association between ‘follow‐up adherence’ and ‘treatment with curative intent’. We classified patients who had on average less than 1 CEA test per year and on average less than 0.5 imaging test per year as having less follow‐up than the guideline recommended. This would address potential misclassification into ‘less than guideline’ in situations where a patient has for example 1 CEA‐test in 9 months and could have had a second test in month 11 or 12 (if they were followed longer e.g. if no recurrence was found). All analyses were performed with R version 4.0.3.

### 2.5 Multiple Imputation

Multiple imputation was used to reduce potential bias due to missingness in the variables number of comorbidities and differentiation grade. We assumed that the data was missing at random (MAR) based on expert judgment, on how data was collected by data managers from the NCR and the lack of discernible correlations between the missing data and the observed data [12]. Multivariate imputation by chained equations was performed using the “mice” package in R. We created 10 imputed datasets with 20 iterations each. The logistic regression analyses and mediation analyses were performed on these datasets. Results were pooled according to Rubin's rule.

## 3 Results

A total of 430 patients were included in this study, after applying the exclusion criteria to the 500 sampled individuals (Figure S1). Median age of the patients was 68 years (Q_1_–Q_3_: 62–75) and the majority was male (62.1%), had their primary tumor in the colon (58.6%) and were diagnosed with stage III (71.6%) (Table 1). Median follow‐up time until recurrent disease was 15.7 months (Q_1_–Q_3_: 10.7–23.6).

**TABLE 1:** Population characteristics.

	Total (*n* = 430)
Age, median (Q1–Q3)	68.0 (62.0–75.0)
Sex, *n* (%)	
Male	267 (62.1)
Female	163 (37.9)
Site, *n* (%)	
RCC	117 (27.2)
LCC	135 (31.4)
RC	178 (41.4)
Disease stage, *n* (%)	
II	122 (28.4)
III	308 (71.6)
No. of comorbidities	
0	211 (49.1)
1	107 (24.9)
≥ 2	65 (15.1)
Unknown	47 (10.9)
Tumor differentiation, *n* (%)	
Poor	7 (1.6)
Moderate	329 (76.5)
Well	51 (11.9)
Unknown	43 (10.0)
Number of evaluated lymph nodes, median (Q1–Q3)	16.0 (12.0–22.0)
Adjuvant chemotherapy, *n* (%)	
No	213 (49.5)
Yes	217 (51.5)

### 3.1 Follow‐Up Adherence

**FIGURE 1:**
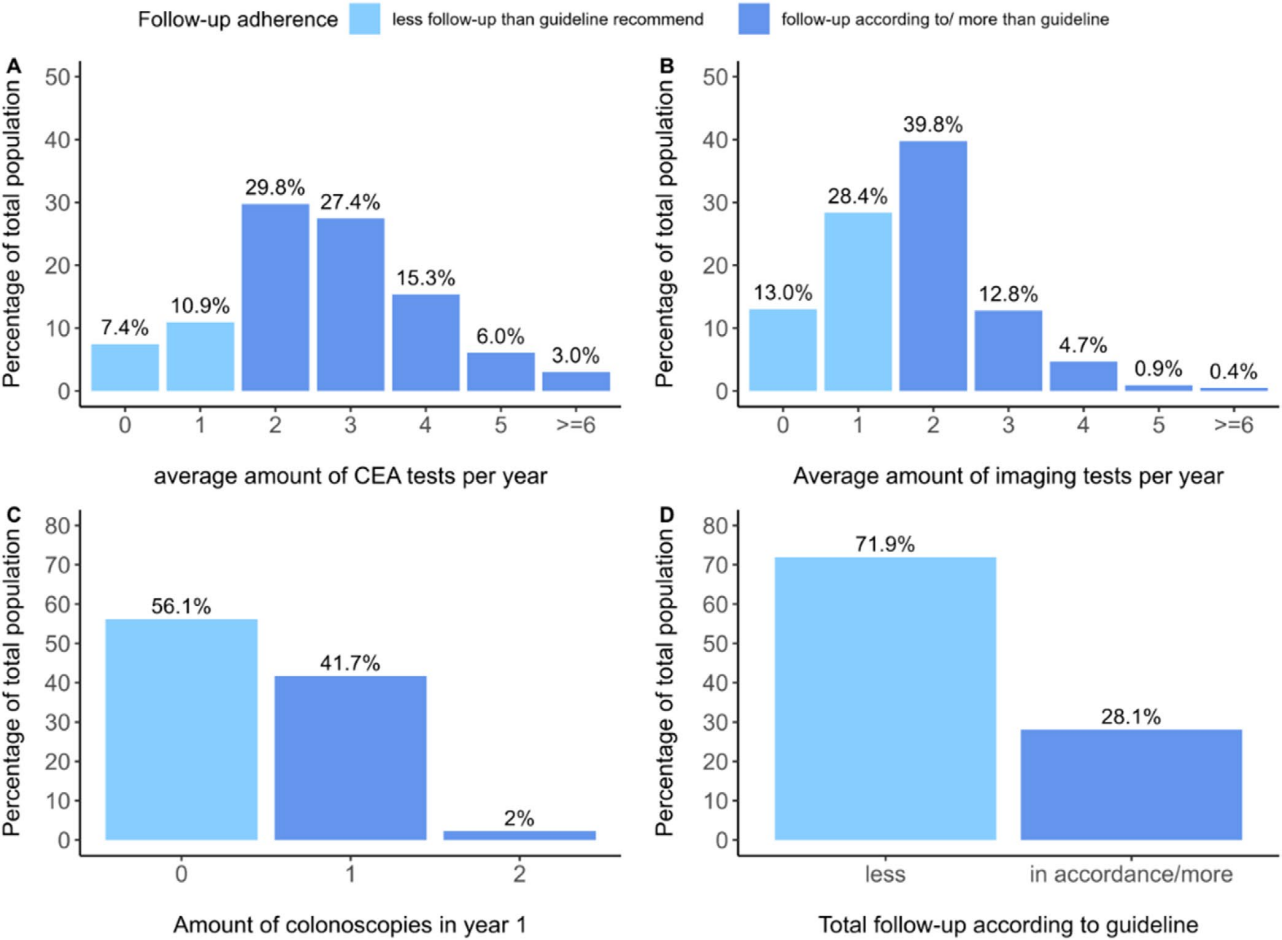
Follow‐up adherence for (A) CEA measurements, (B) imaging tests, (C) colonoscopies and (D) all three modalities combined. Note that histogram C and D represent a subpopulation (*n* = 228) of patients who developed a recurrence after 15 months.

Follow‐up adherence is shown in Figure 1. In total, 18.3% of the patients had fewer CEA measurements than recommended by the guideline. For the imaging, 41.4% of the patients had fewer scans than recommended by the guideline. A total of 202 patients experienced a recurrence within 15 months. Consequently, they were excluded from the adherence assessment of colonoscopy, resulting in a subset of 228 patients. For this subset of patients, 56.1% did not undergo a colonoscopy according to the guideline, that is, between 9 to 15 months after surgery. In total, 28.1% of the patients had CEA, imaging, and colonoscopy according to or more frequently than the guideline. Most patients (71.9%) had less follow‐up than the guideline recommended for at least one modality (e.g., CEA, imaging, or colonoscopy). When considering all three modalities simultaneously, 26 patients (10.0%) received less follow‐up than the guideline recommended. File S2 provides a more detailed description of the adherence across the three modalities.

### 3.2 Factors Associated With Receiving Less Follow‐Up Than the Guideline

Factors associated with having fewer CEA measurements, imaging, and colonoscopies than the guideline recommended are shown in Table 2. Patients aged 75 years or older (OR = 2.4; 95% CI: 2.0–2.8), having ≥ 2 comorbidities (OR = 2.0; 95% CI: 1.6–2.5) and patients having received post‐operative systemic therapy (OR = 1.4; 95% CI: 1.03–1.8) had fewer CEA tests than recommended by the guideline. Furthermore, patients with colon cancer (OR = 0.71; 95% CI: 0.57–0.89) and patients with good/moderate differentiation grade (OR = 0.47; 95% CI: 0.38–0.58) are less likely to have fewer CEA tests than recommended by the guideline.

**TABLE 2:** Parameter estimates for variables associated with receiving less follow‐up.

	CEA‐tests			Imaging			Colonoscopy		
	OR	95% CI	*p*	OR	95% CI	*p*	OR	95% CI	*p*
Site (colon vs. rectum)	**0.71**	**[0.57–0.89]**	**0.003**	**1.45**	**1.26–1.65**	**< 0.001**	**0.72**	**0.54–0.97**	**0.032**
Age (≥ 75 vs. < 75)	**2.39**	**[2.02–2.83]**	**< 0.001**	**1.26**	**1.12–1.41**	**< 0.001**	**1.79**	**1.38–2.32**	**< 0.001**
Pathological stage (III vs. II)	0.86	[0.71–1.04]	0.129	0.90	0.80–1.01	0.076	0.83	0.68–1.01	0.066
No. of comorbidities (1 vs. 0)	0.87	[0.71–1.06]	0.162	0.96	0.86–1.06	0.413	1.12	0.87–1.45	0.389
No. of comorbidities (≥ 2 vs. 0)	**2.02**	**[1.62–2.53]**	**< 0.001**	**0.69**	**0.59–0.82**	**< 0.001**	**2.74**	**1.74–4.32**	**< 0.001**
Tumor differentiation (good/moderate vs. poor/no)	**0.47**	**[0.38–0.58]**	**< 0.001**	1.11	0.93–1.31	0.237	1.18	0.75–1.85	0.473
Adjuvant systemic chemotherapy (yes vs. no)	**1.4**	**[1.03–1.79]**	**0.029**	**1.24**	**1.05–1.47**	**0.013**	**0.49**	**0.35–0.69**	**< 0.001**

Statistically significant associations are highlighted in bold.

Forf imaging, colon cancer (OR = 1.5; 95% CI: 1.3–1.7), age 75 years or older (OR = 1.3; 95% CI: 1.1–1.4) and adjuvant systemic chemotherapy (OR = 1.2; 95% CI: 1.05–1.5) are associated with receiving fewer imaging tests than recommended by the guideline. Patients having ≥ 2 comorbidities are however less likely to have fewer imaging than the guideline compared to patients without comorbidities (OR = 0.69; 95% CI: 0.59–0.82).

Lastly for the colonoscopy, patients of age 75 years or older (OR = 1.8; 95% CI: 1.4–2.3) and patients having two or more comorbidities (≥ 2 vs. 0: OR = 2.7; 95% CI: 1.7–4.3) are more likely to not receive a colonoscopy. Colon cancer patients (OR = 0.72; 95% CI: 0.54–0.97) and patients who received post‐operative systemic chemotherapy (OR = 0.49; 95% CI: 0.35–0.69) are less likely to not receive a colonoscopy.

### 3.3 Follow‐Up Adherence and Recurrence Detection

**FIGURE 2:**
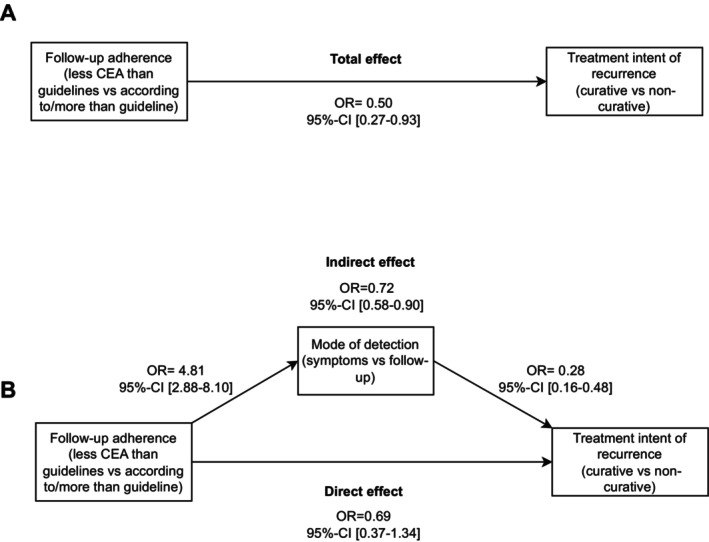
Relationship between CEA adherence and treatment intent of the recurrence (A), mediated by mode of detection (B).

In total, 109 (25.3%) of the patients had an interval‐recurrence, thus detected through symptoms. Patients who had less CEA tests than the guideline recommended had 4.8 times higher odds (95% CI: 2.9–8.1) to have their recurrence detected through symptoms instead of a regular follow‐up test compared to patients who had their CEA follow‐up according to guideline (Figure 2). Patients who had their recurrence detected through symptoms instead of follow‐up, had 0.3 times higher odds (95% CI: 0.2–0.5) to have their recurrence treated with curative intent compared to patients who had their recurrence detected by follow‐up (Figure 2).

The indirect effect from CEA adherence to treatment with curative intent was OR = 0.7 (95% CI: 0.6–0.9) (Table 3). Thus, the average causal mediation effect (ACME) was statistically significant (*p* = 0.003). The direct effect of CEA adherence on treatment with curative intent was OR = 0.7, (95% CI: 0.4–1.3), which was not significant (*p* = 0.27) (Table 3). Thus, the effect of CEA follow‐up adherence on the likelihood of a recurrence being treated with curative intent was fully mediated via the mode of detection. Both for the imaging and colonoscopy tests, the total effects of the relationship between follow‐up adherence and treatment with curative intent were insignificant (*p* > 0.05) and the effect‐sizes were small. Also, there was no significant mediation in the relationship (Table 3, Figures S2 and S3). Results of the sensitivity analyses for CEA adherence and imaging adherence with a less strict cut‐off value are shown in Table S1. The total effect (OR = 0.37; 95% CI: 0.15–0.95) and indirect effect (OR = 0.69; 95% CI: 0.53–0.92) of the association between receiving fewer CEA tests than the guideline and receiving treatment with curative intent remained significant with a stronger effect size than in the main analysis. The results for the association between imaging adherence and curative treatment remained insignificant (*p* > 0.05).

**TABLE 3:** Relationship between guideline adherence and receiving treatment with curative intent, mediated by mode of detection.

	CEA‐tests			Imaging			Colonoscopy		
	OR	95% CI	*p*	OR	95% CI	*p*	OR	95% CI	*p*
Total effect *adh + mod➔ treat* ^a^	**0.50**	**[0.27–0.93]**	**0.029**	0.90	0.58–1.39	0.66	0.85	0.47–1.55	0.673
Indirect effect *adh **➔** mod **➔** treat* ^a^	**0.72**	**[0.58–0.90]**	**0.003**	1.05	0.95–1.16	0.32	0.96	0.83–1.13	0.650
Direct effect *adh**➔** treat* ^a^	0.69	[0.36–1.37]	0.27	0.86	0.56–1.31	0.48	0.87	0.48–1.55	0.653

Abbreviations: adh = adherence (less vs. according to/more), mod = mode of detection recurrence (symptomatic vs. scheduled follow‐up), treat = treatment (curative intent vs. palliative intent).

^a^ Corrected for age and No. of comorbidities. Statistically significant associations are highlighted in bold.

## 4 Discussion

This study showed that almost half of the patients had fewer imaging and no colonoscopy according to the guideline. Roughly 1 out of 5 patients had fewer CEA tests than recommended by the guideline. Receiving less follow‐up was associated with tumor site, age, comorbidities and post‐operative systemic chemotherapy. Lastly, patients who had fewer CEA tests than the guideline recommended, were more likely to have a symptomatic detection of a recurrence and were less likely to receive curative treatment for their recurrence. This relationship between adherence and curative treatment was not found for the imaging nor the colonoscopy modalities.

Few population‐based studies have assessed guideline adherence for CRC follow‐up, potentially due to the extensive effort to collect follow‐up data. One recent systematic review showed that guideline adherence was highest for colonoscopy (70%, 95% CI: 67%–73%), followed by imaging (63%, 95% CI: 47%–80%) and CEA tests (54%, 95% CI: 47%–80%) [11]. In our study, adherence was the highest for CEA tests (81.7%) followed by imaging (58.6%) and colonoscopy (43.9%). The majority of the studies included in the review were cross‐sectional and conducted in Canada or the United States. Differences between healthcare systems in different countries as well as in patient awareness, physician recommendations, cultural beliefs, and socioeconomic factors make comparison difficult. A potential explanation for the low colonoscopy adherence in our study is the low risk of a locoregional recurrence compared to the risk of developing distant metastases, as a result of which colonoscopy could be seen by physicians as a less important part of the follow‐up [13, 14, 15]. Also, colonoscopy is perceived as an unpleasant procedure by patients, which could also contribute to low adherence rate if the patient's preferences for follow‐up are taken into account [16]. Lastly, it is also possible that colonoscopies were scheduled later in the follow‐up based on physician‐ and patient preferences.

The current study found several risk factors for receiving less follow‐up than the guideline recommended. First, colon cancer patients were more likely to undergo CEA testing and a colonoscopy, but less likely to undergo imaging compared with rectal cancer patients. Also, patients with two or more comorbidities were more likely to undergo imaging but less likely to undergo CEA‐tests and colonoscopies, compared to patients without comorbidities. Rectal cancer patients and patients with multiple comorbidities have a higher recurrence risk [17, 18], which could have been considered by physicians. Literature suggests a greater sensitivity in imaging tests such as CT‐scans than in CEA measurements [19, 20]. It could be hypothesized that these patients may have had more imaging to substitute CEA‐tests and the colonoscopy at year 1. Age ≥ 75 years was associated with receiving less follow‐up than the guideline recommended. Literature shows that being of older age is related to receiving less treatment and follow‐up [21, 22], potentially because of the wish not to be treated anymore when a recurrence is detected. Lastly, it must be noticed that adherence to the guideline is inevitably associated with the potential preferences and needs of patients themselves. Therefore, non‐adherence to follow‐up is not necessarily undesirable clinical practice if patients are involved in the decision‐making process.

Several systematic reviews concluded that more intensive follow‐up increases the detection of asymptomatic recurrences [8, 9, 23, 24]. Two important studies within these reviews are the COLOFOL‐ and FACS trials [25, 26]. In the COLOFOL trial, the high‐frequency group received imaging and CEA‐tests at 6, 12, 18, 24, and 36 months after surgery. The FACS trial defined intensive follow‐up as receiving tests according to the guideline in place in our study, while the minimum follow‐up group only received testing if symptoms occurred. The COLOFOL and FACS trial concluded that patients who receive an intensive follow‐up schedule were more likely to be eligible for curative treatment [25, 26]. This is in accordance with our finding that patients who developed a recurrence and had less CEA measurements than guideline were more likely to have their recurrence detected by symptoms instead of follow‐up testing and were less likely to receive curative treatment. However, for imaging and colonoscopy no such relation was found. This can be seen as support for the revision of the Dutch guideline at the end of 2020, in which the role of CEA has increased compared to the role of imaging.

Additionally, these findings can be used to explore the possibilities for remote follow‐up since CEA measurements can be performed at the general practitioner or even at home [27, 28]. Current trials, such as the FUTURE and DISTANCE studies, are investigating whether intensive, in‐hospital postoperative surveillance strategies remain appropriate from both a patient well‐being and societal standpoint [29, 30]. These home‐based follow‐up trials primarily focus on CEA testing. Our study indicates that adherence to CEA testing was the highest, which may suggest a greater acceptance and support for this type of monitoring. It is likely that in‐hospital, specialist‐led colorectal cancer follow‐up will no longer be the only viable and sustainable model in the near future. A more modern, home‐based surveillance approach could offer advantages in terms of both patient quality of life and healthcare costs.

A strong aspect of this study is the longitudinal design in which adherence to the guideline is assessed based on population based observational data. This provides a more accurate reflection of general clinical practice than trials and is less prone to bias compared to adherence studies based on surveys. However, there are some limitations that should be mentioned. First, the study contains no qualitative information about the reason behind deviation from the guideline. On the individual level, physicians can decide on patient‐centred care delivery and therefore tailor the follow‐up schedule to the patients' needs and preferences. A second limitation is that we could not distinguish additional diagnostic testing from tests performed in the regular follow‐up. When CEA values slightly increase, or when a CT‐scan or ultrasound is inconclusive, a physician can decide to monitor the patient more closely by repeating the tests in a shorter interval. For this reason, we combined the group ‘according to the guideline’ and ‘more than the guideline’ in the analyses and compared it to the group ‘less follow‐up than the guideline’. Also, It is difficult to discern whether CT‐scans are ordered specifically for CRC surveillance or for the management of other comorbidities. This ambiguity can lead to an overestimation in adherence to imaging. Thirdly, the categorization of ‘less CEA or imaging than the guideline’ could be perceived as strict, especially compared to the categorization of the colonoscopy adherence. We acknowledge that some patients could potentially have developed a recurrence shortly before receiving their scheduled CEA‐test or CT‐scan, which may have placed them in the ‘less follow‐up than the guideline’ category. Nevertheless, our sensitivity analysis in which we used a less strict classification for ‘according or more follow‐up than the guideline’ showed that the associations between adherence and curative treatment remain similar in strength for imaging and the associations for the CEA‐tests become stronger. Therefore, we argue that this did not impact our results.

It is important to assure that providing less follow‐up than recommended is based on patients' needs and the potential for health benefits, rather than unintended differences in health care provision. The relevance of follow‐up is based on the assumption that finding a recurrence earlier by follow‐up leads to better chances of survival because the recurrence is detected at an earlier stage and can therefore still be treated curatively. It should be noted that although more intensive follow‐up could increase the number of patients undergoing surgery with curative intent, its effect on overall survival is difficult to determine. This challenge arises from the potentially small numerical differences between trial arms with different follow‐up schedules. Trials require a large sample size to detect any significant differences in overall survival and are not being powered accordingly. Additionally, it's probable that patients with biologically favorable characteristics who develop metastases may still experience positive outcomes even without early detection. Further research should therefore focus on the survival benefit by adhering to the guideline in place, taking into consideration that adherence is influenced by age and comorbidities, and employing causal inference methods to better understand these relationships.

## 5 Conclusion

This population‐based study in the Netherlands revealed varied adherence to the follow‐up guideline among patients. Most patients adhered well to CEA tests but did not receive the recommended imaging tests and colonoscopies. Tumor site, age, number of comorbidities and adjuvant systemic chemotherapy were associated with follow‐up adherence. Notably, receiving fewer CEA measurements than recommended was linked to recurrences being detected through symptoms rather than follow‐up visits, and was associated with not undergoing curative treatment. This is in line with the shift toward prioritizing CEA measurement over imaging in recent guideline adaptations.

## Author Contributions

**Tara C. Boute:** formal analysis (lead), methodology (lead), project administration (lead), software (equal), validation (equal), visualization (lead), writing – original draft (lead), writing – review and editing (lead). **Rik van Eekelen:** formal analysis (supporting), methodology (supporting), software (equal), validation (equal), writing – review and editing (supporting). **Marloes A. G. Elferink:** data curation (lead), investigation (lead), resources (lead), writing – review and editing (supporting). **Lissenberg Witte Birgit I:** methodology (supporting), software (supporting), writing – review and editing (supporting). **Johannes H. W. de Wilt:** conceptualization (supporting), funding acquisition (supporting), writing – review and editing (supporting). **Geraldine R. Vink:** conceptualization (supporting), funding acquisition (lead), writing – review and editing (supporting). **Marjolein J. E. Greuter:** conceptualization (lead), methodology (equal), project administration (equal), supervision (lead), writing – review and editing (equal). **Veerle M. H. Coupé:** conceptualization (lead), funding acquisition (lead), methodology (equal), supervision (lead), writing – review and editing (equal).

## Disclosure

The authors have no conflicts of interest to disclose.

## Ethics Statement

The present study was approved by the privacy review board of the NCR. According to the Central Committee on Research involving Human Subjects (the Hague, Netherlands), NCR studies do not require ethics approval as all data are anonymized and deidentified.

## Consent

The Netherlands Cancer Registry requires no informed consent of patients to collect data according to Dutch law.

## Conflicts of Interest

The authors declare no conflicts of interest.

## Supporting information


Data S1.

## Data Availability

The data that support the findings of this study are available from the Netherlands. Comprehensive Cancer Organization (IKNL). Restrictions apply to the availability of these data, which were used under license for this study. Data are available from the corresponding author, T.C., with the permission of IKNL.
