# Supplementary material for: Follow‐Up Adherence After Treatment With Curative Intent for Stage II and III Colorectal Cancer Patients
Source: Cancer Med. 2025 Feb 27;14(5):e70667. doi: 10.1002/cam4.70667 (PMC11865713; doi:10.1002/cam4.70667)
Supplement: Supplementary file 1 — Data S1. [file CAM4-14-e70667-s001.docx]

**Supplementary File 1: mediation analyses**


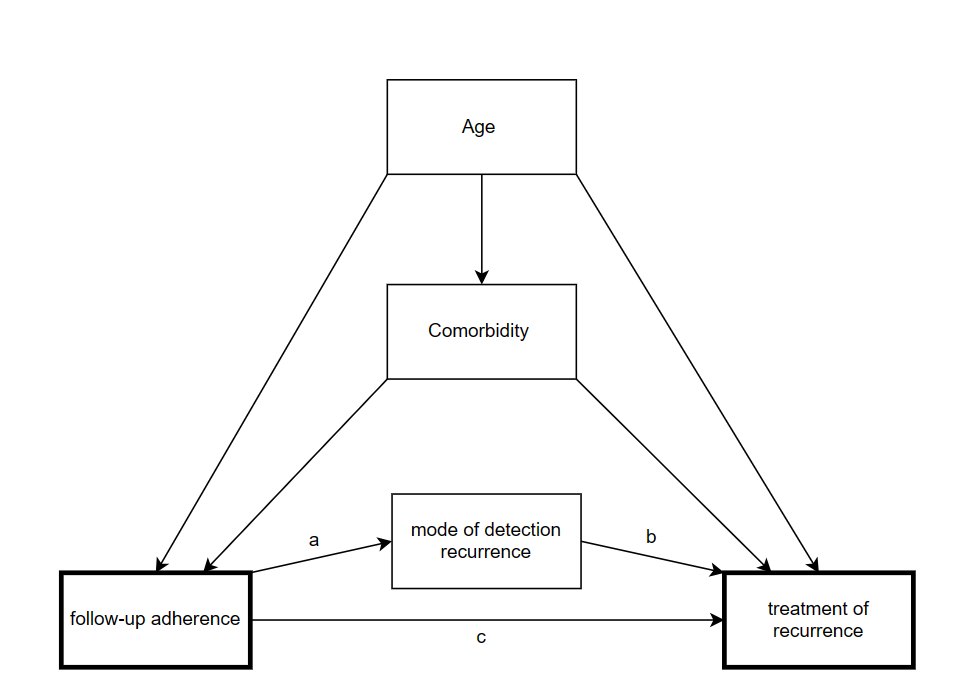


*Directed Acyclic Graph of relationship between follow-up adherence and treatment of recurrence*

**Paths a, b, and c:**

**Path a (follow-up adherence -> mode of detection):** This path represents the relationship between follow-up adherence (independent variable) and the mode of detection (mediator variable). It shows how likely it is for an individual to have his recurrence detected through symptoms instead of regular follow-up when he adheres to the prescribed follow-up schedule.

**Path b (mode of detection -> treatment of recurrence):** This path represents the relationship between the mode of detection (mediator variable) and the treatment of recurrence (dependent variable). It reflects whether the mode of detection (symptomatic vs. regular follow-up) influences the possibility of treatment with curative intent of the recurrence (curative vs. non-curative).

**Path c (follow-up adherence -> treatment of recurrence):** This path represents the direct relationship between follow-up adherence (independent variable) and treatment of recurrence (dependent variable). It indicates whether adhering to follow-up leads to a certain type of treatment for recurrence, irrespective of the mode of detection.

**Direct, Indirect, and Total Effects:**

**Direct Effect (c'):** The direct effect of follow-up adherence on treatment of recurrence (c') is the effect that remains after accounting for the influence of the mediator (mode of detection). It is the relationship between follow-up adherence and treatment of recurrence that is not mediated by the mode of detection.

**Indirect Effect (ab):** The indirect effect of follow-up adherence on treatment of recurrence through the mode of detection (ab) represents the effect that operates through the mediator variable. It captures how much of the relationship between follow-up adherence and treatment of recurrence is explained by the mode of detection.

**Total Effect (c):** The total effect of follow-up adherence on treatment of recurrence (c) includes both the direct effect (c') and the indirect effect (ab). It represents the overall impact of follow-up adherence on treatment of recurrence, regardless of whether it operates through the mode of detection or not.

**Supplementary Figure 1: Flowchart of inclusion and exclusion of patients**

**
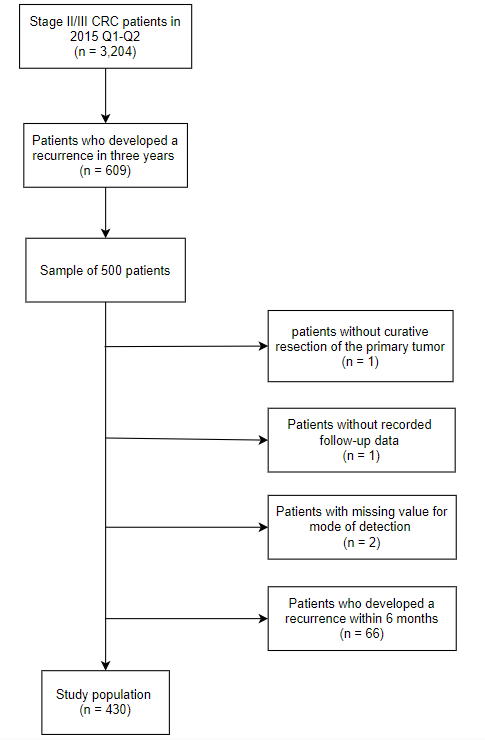
**

**Supplementary Figure 2: relationship between Imaging adherence and treatment of the recurrence, mediated by mode of detection**


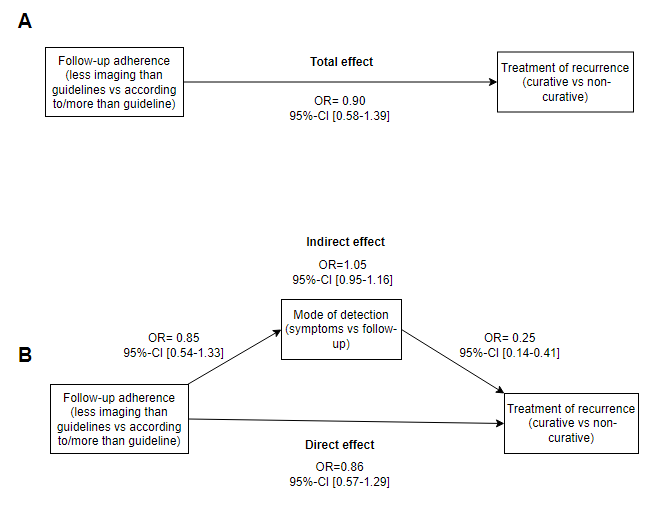


**Supplementary Figure 3: relationship between colonoscopy adherence and treatment of the recurrence, mediated by mode of detection**


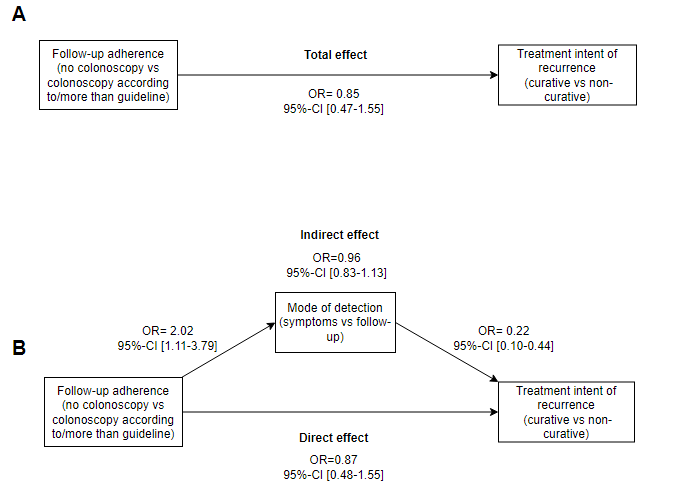


**Supplementary Table 1**: Sensitivity analysis for the relationship between CEA and imaging guideline adherence and receiving curative treatment, mediated by mode of detection

|  |  |  | CEA-tests |  |  |  | Imaging |  |
| --- | --- | --- | --- | --- | --- | --- | --- | --- |
|  |  | OR | 95%-CI | p-value |  | OR | 95%-CI | p-value |
|  |  |  |  |  |  |  |  |  |
| Total effect *adh + mod🡪 treat** |  | **0.37** | **[0.15-0.95]** | **0.040** |  | 1.09 | 0.53-2.25 | 0.823 |
| Indirect effect  *adh 🡪 mod 🡪 treat** |  | **0.69** | **[0.53-0.92]** | **0.009** |  | 0.85 | 0.69-1.06 | 0.155 |
| Direct effect *adh🡪 treat** |  | 0.53 | [0.07-4.72] | 0.557 |  | 1.28 | 0.59-2.73 | 0.533 |
|  |  |  |  |  |  |  |  |  |

adh = adherence (less vs according to/more), mod = mode of detection recurrence (symptomatic vs scheduled follow-up), treat = treatment (curative vs. palliative)

* corrected for age and Charlson Comorbidity Index

CEA less than the guideline was defined as less than 1.0 tests on average per year

Imaging less than the guideline was defined as less than 0.5 tests on average per year

**Supplementary File 2**: Patient adherence patterns in 2x2 Contingency Tables

|  | Less imaging than GL | Imaging according to GL |
| --- | --- | --- |
| Less CEA than GL | **47**  *Row: 59%*  *Column: 26%* | **32**  *Row: 41%*  *Column: 13%* |
| CEA according to GL | **131**  *Row: 37%*  *Column: 74%* | **220**  *Row: 63%*  *Column: 87%* |

|  | Less colonoscopies than GL | Colonoscopies according to GL |
| --- | --- | --- |
| Less CEA than GL | **33**  *Row: 80%*  *Column: 26%* | **8**  *Row: 20%*  *Column: 8%* |
| CEA according to GL | **95**  *Row: 51%*  *Column: 74%* | **92***Row: 49%*  *Column: 92%* |

|  | Less colonoscopies than GL | Colonoscopies according to GL |
| --- | --- | --- |
| Less imaging than GL | **64**  *Row: 66%*  *Column: 50%* | **33**  *Row: 34%*  *Column: 33%* |
| Imaging according to GL | **64**  *Row: 49%*  *Column: 50%* | **67**  *Row: 51%*  *Column: 67%* |
